# Coinfection of *Babesia* and *Borrelia* in the Tick *Ixodes ricinus*—A Neglected Public Health Issue in Europe?

**DOI:** 10.3390/pathogens13010081

**Published:** 2024-01-17

**Authors:** Thomas G. T. Jaenson, Jeremy S. Gray, Per-Eric Lindgren, Peter Wilhelmsson

**Affiliations:** 1 Evolutionary Biology Centre, Department of Organismal Biology, Uppsala University, Norbyvägen 18d, SE-752 36 Uppsala, Sweden; thomas.jaenson@ebc.uu.se; 2 UCD School of Biology and Environmental Science, University College Dublin, D04 N2E5 Dublin, Ireland; jeremy.gray@ucd.ie; 3 Division of Inflammation and Infection, Department of Biomedical and Clinical Sciences, Linköping University, SE-581 83 Linköping, Sweden; per-eric.lindgren@liu.se; 4 Department of Clinical Microbiology, Region Jönköping County, SE-551 11 Jönköping, Sweden

**Keywords:** *Babesia*, *Borrelia*, babesiosis, coinfection, *Ixodes ricinus*, Lyme borreliosis, Sweden

## Abstract

*Ixodes ricinus* nymphs and adults removed from humans, and larvae and nymphs from birds, have been analysed for infection with *Babesia* species and *Borrelia* species previously in separately published studies. Here, we use the same data set to explore the coinfection pattern of *Babesia* and *Borrelia* species in the ticks. We also provide an overview of the ecology and potential public health importance in Sweden of *I. ricinus* infected both with zoonotic *Babesia* and *Borrelia* species. Among 1952 nymphs and adult ticks removed from humans, 3.1% were PCR-positive for *Babesia* spp. Of these *Babesia*-positive ticks, 43% were simultaneously *Borrelia*-positive. Among 1046 immatures of *I. ricinus* removed from birds, 2.5% were *Babesia*-positive, of which 38% were coinfected with *Borrelia* species. This study shows that in *I. ricinus* infesting humans or birds in Sweden, potentially zoonotic *Babesia* protozoa sometimes co-occur with human-pathogenic *Borrelia* spp. Diagnostic tests for *Babesia* spp. infection are rarely performed in Europe, and the medical significance of this pathogen in Europe could be underestimated.

## 1. Introduction

### 1.1. Aims

The main aim of this paper is to discuss the significance of coinfections of Babesia spp. and Borrelia spp. in ticks in Europe. The occurrence and interaction of the relevant pathogens are first reviewed and related to earlier data on zoonotic *Babesia* species in Europe, their mammal reservoirs and tick vectors, co-occurring microorganisms in ticks, coinfections of ticks with more than one tick-borne pathogen species, and human disease caused by *Babesia*+*Borrelia* coinfections. Then, studies in Sweden investigating coinfections of *Borrelia* spp. and *Babesia* spp. in ticks removed from humans or birds are presented. The Discussion section deals with *Babesia*+*Borrelia* coinfections in humans, the possibility that there may be undetected coinfections, the possibility that some symptoms in persistent LB cases may be due to accompanying babesiosis, and the possible importance of transfusion blood infected with tick-borne pathogens including *Babesia* parasites. After the concluding remarks, there is a section on suggested research needs.

### 1.2. Background

It is generally accepted that most hosts are coinfected most of the time with two or more parasite species [1]. In this context, parasites may be viruses, bacteria, protozoa, helminths, and arthropods. The different parasite species may be mutually beneficial due to effects on the host’s immune system, or they may be antagonistic to one or more of the other parasites due to, for instance, resource competition [1]. An ongoing infection can have strong effects on a host’s risk of contracting further infections, even stronger than more obvious reasons such as age and nutritional state [1]. 

Telfer and collaborators [1] investigated in detail the interactions among members of a microparasite community infecting field voles (*Microtus agrestis*) and showed that infection with *Babesia microti, Bartonella* spp., or *Anaplasma phagocytophilum* increased the risk of infection with other species. Decreased susceptibility to other pathogens was also recorded; for example, *Ba. microti* strongly reduced susceptibility to *Bartonella* infection. This effect was particularly significant in rodents chronically infected with *Ba. microti* [1]. An important conclusion of this study was that even if strong associations between one particular infection and host fitness are found, attributing the effect to that parasite alone may hide the effects caused by other parasite species in the community [1].

*Ba. microti* is an important zoonotic tick-borne pathogen in the United States and is transmitted by the same vector, *Ixodes scapularis*, that transmits Lyme disease (Lyme borreliosis, LB), caused by the spirochaete, *Borrelia burgdorferi*. These two pathogens have been shown to have significant interactions, both at the patient level [2] and within the tick and reservoir hosts [3,4]. The increased incidence and geographic distribution of human babesiosis in the United States have been preceded by significantly increased incidence and geographic range expansion of LB in the last 25 years [5,6,7,8]. 

Some aspects of the epidemiology and ecology of tick-borne infections in Sweden, particularly LB [9], and even babesiosis, have certain similarities to those of LB and babesiosis in the United States. However, most of the information about the public health aspects, epidemiology, and ecology of zoonotic babesiosis comes from studies on *Ba. microti* in the United States, which appears to differ from that for *Ba. microti* in Europe, where it poses a much smaller public health problem. However, European tick-borne encephalitis (TBE) is transmitted by the same vector, *Ixodes ricinus,* that transmits LB in Europe, and epidemiological data from several studies show that the incidences of these tick-transmitted human diseases have increased during this century [10,11,12,13,14,15,16], accompanied by an increase in the geographic range and abundance of *I. ricinus* [14,17]. We suggest that in Northern Europe, there may also be an increase, although weaker, in the incidence of unconfirmed/undetected human babesiosis cases.

### 1.3. Zoonotic Babesia Species in Europe

*Babesia* species are naturally transmitted by ixodid and a few argasid tick species and infect the erythrocytes of mammals and birds. There are more than 120 described species of *Babesia* [18,19,20,21,22]. The majority have been recorded in mammals, but 16 species have so far been described from birds [21,22]. Relatively few *Babesia* spp. are known to cause disease in humans. *Ba. microti* is responsible for the majority of cases, notably in the United States, and *Babesia divergens* is the main zoonotic pathogen in Europe, with *Babesia venatorum* and a *Babesia crassa*-like organism contributing very rare cases. Zoonotic babesiosis has been reviewed recently by Krause [23], Hildebrandt et al. [24], and Bajer et al. [25], and the disease is not discussed in detail in this paper. To briefly summarise, haemolysis is the initial pathological event in babesiosis, resulting from the release of parasites from erythrocytes following asexual reproduction, potentially causing jaundice, anoxia, and organ failure. Patients present with fever, headache, night sweats, and myalgia, and in heavy infections, haemoglobinuria may be present. Zoonotic genotypes of *Ba. microti* can cause illness in immunocompetent patients, ranging from chronic to acute infections, but *Ba. divergens* and *Ba. venatorum* infections are usually acute, occurring in immunocompromised patients, with splenectomy a high-risk factor. A few recent cases have occurred in immunocompetent patients, and it is probable that asymptomatic cases occur, though without chronicity [26].

Most *Babesia* species (*Babesia* sensu stricto or Clade X) can invade the ovaries of an infected adult female tick so that vertical (transovarial) transmission of the babesiae can take place, potentially rendering her larval offspring already infected when they emerge from the eggs [26,27]. *Ba. divergens* and *Ba. venatorum* are members of this group, and in both cases, the parasites can persist transstadially from larva via nymph to the adult stage without any need for reinfection [27]. In contrast, transovarial transmission does not take place in *Ba. microti* complex species, and the tick vectors acquire the infection when larvae or nymphs feed on infective small mammals with the parasites probably only surviving one moult [26]. Based on recent phylogenetic analysis of 18S rDNA sequences, Goethert [28] concludes that the *Ba. microti* species complex encompasses at least five distinct clades. Parasites from Clade 1, also referred to as *Ba. microti* sensu stricto or US-type, are responsible for nearly all human babesiosis cases worldwide, especially in the United States, where it is relatively common [28]. Although Clade 1 *Ba. microti* parasites occur in Europe, a similar public health problem to that in the United States does not exist in Europe, where only a few autochthonous, zoonotic *Ba. microti* cases and some additional imported cases have been recorded [24]. The Munich strain, which is widespread in Europe, was previously thought to be non-zoonotic [29], but this needs to be reconsidered following reports from Poland and Spain of patients apparently infected with this strain showing non-specific symptoms [24,30,31]. However, it should be noted that according to Goethert [28], many of the early results on *Ba. microti* identity, life cycle traits, etc., are questionable since these investigations were carried out prior to the availability of DNA sequencing technology [28]. 

### 1.4. Animal Reservoirs of Zoonotic Babesia Species in Europe

Cattle are the main vertebrate reservoirs of *Ba. divergens* and roe deer (*Capreolus capreolus*) of *Ba. venatorum*. *Ba. venatorum* [32,33] has also on some occasions been recorded from moose (*Alces alces*) [34,35] and from reindeer (*Rangifer tarandus*) [35]. Roe deer are also the main reservoirs of the non-zoonotic *Babesia capreoli* [36]; indeed, some *Ba. divergens*-like babesiae reported from cervids are likely to be *Ba. capreoli* [35,37]. 

Small rodents (*Apodemus, Microtus, Myodes* spp.) and shrews (*Sorex* spp.) are considered to be the reservoirs of *Ba. microti* in Eurasia [26,38]. Three studies [39,40,41] suggest that some bird species may be reservoir-competent for *Ba. microti*. However, as indicated by Goethert [28], the precise molecular identity of detected sequences in such studies is uncertain. In a study by Wilhelmsson and coworkers at Ottenby Bird Observatory in South-Eastern Sweden, 4601 migratory birds of 65 species were examined for tick infestation [42]; 1102 ticks were available for a molecular analysis of *Babesia* species. Nearly all ticks were immatures of *I. ricinus,* of which 2.5% were positive for one of each of these mammal-associated *Babesia* species: *Ba. venatorum* (58%), *Ba. microti* (38%) and *Ba. capreoli* (4.0%). Owing to the absence of transovarial transmission in the tick population, *Ba. microti* was, as expected, only detected in nymphs, whereas the other two *Babesia* species were found in larvae and *Ba. venatorum* also in nymphs [42]. The fact that only nymphs were positive for *Ba. microti* supports the indication that birds are not reservoirs of this species. Furthermore, positive nymphs attached to birds probably have no epidemiological significance since *Ba. microti* is thought to persist through just one moult [43].

### 1.5. Ixodid Vectors of Zoonotic Babesiae in Europe

The ability of different tick species to act as competent vectors for *Babesia* species of veterinary and medical importance was recently reviewed by Gray et al. [26]. Transmission of *Ba. divergens, Ba. venatorum* and *Ba. microti* by *I. ricinus* has been demonstrated experimentally [27]. *Ba. microti* has been reported in *Ixodes persulcatus* in Eastern Europe [44,45], but Korenberg and coworkers consider *I. persulcatus* an insignificant vector of *Ba. microti* in Russia [46]. In contrast, Zamoto-Niikura et al. [47,48] and Sun et al. [49] regard this tick species as an important vector of *Ba. microti* in Japan and North-Eastern China, respectively. *I. persulcatus* is apparently a vector of *Ba. venatorum* in China [49].

*Ixodes trianguliceps* was previously considered to be the main enzootic vector of *Ba. microti* in Europe [18,50] but, as emphasised by Goethert [28], in early studies, molecular sequencing was not available, so it is not known if the parasites studied were *Ba. microti* of Clade 1 or Clade 3 [50,51,52,53]. The current view is that *I. trianguliceps* is the main vector of the Munich strain [38,53], and since this tick species rarely bites humans, it is unlikely to be a significant vector of human babesiosis. Thus, *I. ricinus* can be considered the main vector of zoonotic *Babesia* species in Europe [26,43]. The prevalence of Babesia spp. in Ixodes ricinus in Northern Europe is shown in Table 1.

### 1.6. Coinfections with Two or More Tick-Borne Pathogens

High coinfection rates of tick-borne pathogens (TBPs) occur in *I. ricinus* in Europe [68,69,70,71]. In a study in France, analysis of 267 questing adult female *I. ricinus* revealed that almost 50% of infected ticks were coinfected and that a few ticks carried up to five TBPs [68]. Patients who had been bitten by ticks in South-Eastern Sweden were shown to exhibit unspecific symptoms which may have been caused by *Bo. burgdorferi* s.l., *A. phagocytophilum*, or TBE virus, singly or in combination [72]. A large-scale study in the Netherlands and Belgium on the circulation of *Babesia* species and exposure of people to these pathogens involved analysis of 8832 nymphal *I. ricinus* for *Babesia* species, *Bo. burgdorferi* s.l, *Neoehrlichia mikurensis, Borrelia miyamotoi* and *A. phagocytophilum* [67]. Coinfection of *Ba. microti* with *Bo. burgdorferi* s.l. and *N. mikurensis* occurred significantly more often than expected by chance, presumably because these TBPs share the same species of small mammal reservoirs. In contrast, *Babesia* Clade X, i.e., *Ba. venatorum* and related species, co-occurred with *A. phagocytophilum* significantly less often than expected [67]. This phenomenon may be related to the (presumed) immunosuppressive activity of *A. phagocytophilum* when occurring together with *Ba. divergens* in cattle [67]. In Latvia, Capligina and coworkers carried out another large-scale country-wide investigation of TBPs in field-collected ticks during 2017–2019 [73]. Analysis of the ticks found that 33.4% carried at least one TBP, and 5.5% of tick samples were PCR-positive for two or three pathogens. Coinfection with three pathogens was detected in 0.55% (21/3840) of *I. ricinus* ticks. A higher overall prevalence of TBPs was observed in *I. ricinus* (34.9%) and *I. persulcatus* (31.6%) than in *Dermacentor reticulatus* (24.2%). The molecular analysis showed the presence in *I. ricinus* nymphs (0.4%) of TBE virus (also in adult ticks); *Ba. microti* in 0.29% of adult males, 1.3% of females and 0% of nymphs; *Ba. venatorum* in 1.4% of males, in 1.2% of females and 0.5% of nymphs; *Ba capreoli* in 0.08% of female *I. ricinus*; and *Ba. canis* in 1.4% of males, 0.8% of females and 0.5% of nymphs. Overall, 15 and 7 tick-borne pathogen species were detected in *Ixodes* spp. and *D. reticulatus* ticks, respectively. Forty percent of male and female adult *I. ricinus* contained at least one TBP. For *I. ricinus* nymphs, this proportion was 22% [73]. 

Cutler and coworkers recently reviewed the subject of coinfections within ticks and their hosts and provided insights into challenges and gaps that should be subject to more intensive research [74]. Coinfection of an individual *Ixodes* tick with one or more *Babesia* species and one or more other TBPs, e.g., species of *Borrelia*, *Anaplasma*, *Ehrlichia*, *Neoehrlichia*, *Rickettsia*, *Francisella* and/or TBEV, has been recorded many times (see for example [11,26,39,67,75,76,77,78,79,80,81,82]). At least five European species in the *Borrelia burgdorferi* s.l. complex are considered human-pathogenic, i.e., *Bo. burgdorferi* sensu stricto (s.s.), *Borrelia afzelii, Borrelia garinii*, *Borrelia bavariensis* and *Borrelia spielmanii* [83], and able to cause LB [84]. Furthermore, possible human pathogens include *Borrelia bisettii*, *Borrelia lusitaniae* and *Borrelia valaisiana. Bo. afzelii* and *Bo. garinii* are the species causing most cases of human LB in Europe and are also the genospecies most often detected in *I. ricinus* and *I. persulcatus* [9]. 

Simultaneous infection of *Babesia* parasites and *Bo. burgdorferi* s.l. bacteria in the Nearctic *I. scapularis* [5] and the Palaearctic *I. ricinus* [85,86] and *I. persulcatus* [87] has been repeatedly reported. Alekseev and coworkers obtained data on infected *I. persulcatus* collected near St. Petersburg, Russia, and suggested that *Ba. microti* may only survive in *I. persulcatus* if the ticks are coinfected with *Borrelia* spp. [87]. Coinfection rates of 1.0–22% for *Ba. microti+Bo. burgdorferi* have been recorded in *I. scapularis* [88]. Both infections can be transmitted simultaneously by ticks concurrently infected with both pathogens.

### 1.7. Babesia+Borrelia Coinfections in Humans

Parveen and Bhanot discuss disease manifestations in humans and laboratory mammals, caused by coinfection with *Ba. microti* and *Bo. burgdorferi* [89]. Several studies from 1983 onwards in the United States showed that *Ba. microti* infections in the white-footed mouse (*Peromyscus leucopus*) are strongly correlated with simultaneous *Bo. burgdorferi* infections [90,91,92]. Thus, a high proportion of humans who are infected with *Ba. microti* are also seropositive for *Bo. burgdorferi* [90,93] or concurrently infected with *Bo. burgdorferi* [94]. Subsequent studies have reached the same conclusions, i.e., that the seroprevalence of *Ba. microti* is highest (>50%) among people with active or previous *Bo. burgdorferi* infection [90,93]. Coinfections of *Ba. microti+Bo. burgdorferi* in humans in the United States seem to be more severe and of longer duration compared to a monoinfection with either *Ba. microti* or *Bo. burgdorferi* [95,96]. Coinfected patients have influenza-like symptoms twice as often as patients with only LB [2]. Splenomegaly is also more common in coinfected patients [2,90]. The longer duration with persistent symptoms, particularly extreme fatigue, in coinfected patients may partly be explained by a delay in diagnosis and inadequate treatment of babesiosis [2,90]. In the United States, a bite by an *Ixodes* tick coinfected with *Borrelia* spirochaetes and a human-pathogenic *Babesia* species may therefore potentially cause serious disease and death in asplenic or otherwise immunocompromised humans, including the elderly with comorbidities. 

In support of the above observations, there is evidence from studies on laboratory mice (*Mus musculus*) simultaneously infected with *Bo. burgdorferi* and *Ba. microti* that the latter suppresses the immune response, resulting in enhanced LB symptoms [97]. In another mouse model, Bhanot and Parveen found that the clinical symptoms characteristic of LB increased, whereas the *Ba. microti* parasitaemia was reduced [98]. In both mice and humans, therefore, coinfection of *Ba. microti* and *Borrelia* seems to increase symptoms of LB. It should be emphasised that most of these data on severity of disease symptoms in coinfected patients or laboratory animals refer to studies on pathogen genotypes of American origin. 

In Europe *I. ricinus* ticks can be simultaneously infected with potentially human-pathogenic LB *Borrelia* species and potentially human-pathogenic *Babesia* species such as *Ba. venatorum*, *Ba. divergens* and *Ba. microti*. Regarding *Ba. divergens/Ba. venatorum/Ba. microti+Bo. burgdorferi* s.l. coinfections in humans in Europe, similar disease incidences as for *Ba. microti+Bo. burgdorferi* in the United States do, in general, not occur [90,99]. For instance, one study at the Medical University of Bialystok, Poland, in 2002 involved 74 patients who were diagnosed with infections of *Bo. burgdorferi* s.l. or suspected of having LB based on clinical symptoms, but *Babesia* DNA was not found in the blood of any of these patients [99]. In a Slovenian study of febrile illnesses following tick bites in 53 children, the most common aetiology was TBE followed by LB. There was no evidence of acute or past infection with *Ba. microti* [100]. However, in Finland, a fatal case of *Ba. divergens* infection occurred in a 53-year-old man with no history of splenectomy but with a rudimentary spleen and a LB coinfection [101]. Svensson and coworkers investigated the seroprevalence of people in southernmost Sweden and showed that positive IgG titers for at least one of the species *Ba. divergens* and *Ba. microti* were significantly more prevalent (16.3%) in individuals seropositive for *Bo. burgdorferi* s.l. compared to the healthy control group (2.5%) [102]. These results agree with those of Dunn and coworkers in the United States, who showed experimentally that the frequency of *Ba. microti*-infected ticks increased when they were fed on mice that were coinfected with *Bo. burgdorferi* and *Ba. microti* [3].

## 2. Analysis of Coinfection Data from Sweden

### 2.1. Borrelia spp. and Babesia spp. in Ixodes ricinus

Based on four of our previously published investigations [42,85,103,104], we here describe and analyse the *Babesia*+*Borrelia* coinfection patterns in *I. ricinus* ticks which had been attached to humans or to birds. One of these investigations concerned *I. ricinus* ticks that had been attached to humans. These ticks had been analysed for species composition and prevalence of *Borrelia* spp. [104] and *Babesia* spp. [85]. Two other investigations concerned *I. ricinus* ticks that had been removed from birds and subsequently analysed for the presence and prevalence of *Borrelia* spp. and the TBE virus [103] and *Babesia* spp. [42]. 

Fisher’s exact test was applied to compare the *Babesia*+*Borrelia* coinfection prevalence in ticks that had been attached to humans versus that in ticks that had been attached to birds. We used prevalence data for the species *Ba. divergens, Ba. microti* and *Ba. venatorum*; thus, for this analysis, we omitted our data for the non-zoonotic species *Ba. capreoli*. All ticks in the analyses belonged to *I. ricinus*. Regarding ticks that had been attached to humans, we used only *Babesia* and *Borrelia* infection data of adult and nymphal ticks. Thus, we excluded data obtained for larval ticks, of which only one tick larva (1/86; 1.2%) was positive (for *Ba. capreoli*). Regarding *I. ricinus* removed from birds, no adult tick specimen was collected, so the analyses regarding bird-infesting ticks are based on subadult (immature) ticks, i.e., larvae and nymphs.

#### 2.1.1. Prevalence of *Borrelia* Species in *I. ricinus* Removed from Humans

A total of 2006 *I. ricinus* ticks were collected and analysed, as described in more detail in [104]. Briefly, as presented in Table 1 of that study, 496 adult female ticks and 1510 nymphal ticks were removed after being attached to adult humans in Sweden or on the Åland Islands, Finland, from May to November 2008 and 2009 (Table 1 in [104]). To detect and quantify *Borrelia* 16S rRNA, genus-specific primers were used in a LUX real-time PCR assay as described in [105]. To determine the *Borrelia* species in the real-time PCR-positive samples, a nested PCR assay was applied followed by sequencing, as described in [105]. 

Among *I. ricinus* removed from humans, the *Borrelia* prevalence was 35% in 496 adult female ticks and 24% in 1510 nymphal ticks (Table 1 in [104]). Six species in the *Bo. burgdorferi* s.l. complex and *Bo. miyamotoi* were detected. *Bo. afzelii* was the most prevalent species in both female ticks and nymphs (56% and 38%, respectively). The second most prevalent *Borrelia* species in female ticks and nymphs was *Bo. garinii* (25% and 17%, respectively). The prevalence of the other *Borrelia* species was 9% for *Bo. valaisiana* in adult females; 6% for *Bo. burgdorferi* s.s. in adult females; 3% and 2% for *Bo. miyamotoi* in adult females and nymphs, respectively; and 1% in both adult females and nymphs for *Bo. spielmanii* and *Bo. lusitaniae* [104].

#### 2.1.2. Prevalence of *Babesia* Species in *I. ricinus* Removed from Humans

In total, 1466 (70%) nymphs and 486 (23%) adult *I. ricinus* ticks were analysed for potentially zoonotic *Babesia* species, as described in [85]. The same ticks had previously been analysed for the presence of *Borrelia* spp. [104]. Three *Babesia* species were detected: *Ba. microti* (n = 33), *Ba. venatorum* (n = 27) and *Ba. capreoli* (n = 5); 46% of the *Babesia*-positive ticks also contained *Borrelia* spp. (Table 2) [85] and 3.1% (60/1952) of nymphs and adult ticks were positive for possibly zoonotic *Babesia* spp. (*Ba. capreoli* excluded).

#### 2.1.3. Prevalence of *Borrelia* Species in *I. ricinus* Removed from Birds 

In total, 514 larvae and 549 nymphal *I. ricinus* were analysed for the presence of *Borrelia* species, as described in [103]. Among immature *I. ricinus* ticks removed from birds, the *Borrelia* prevalence was 15.6% (80/514) in larvae and 37.2% (204/549) in nymphs (Table 2 in [103]). Seven *Borrelia* species were identified: *Bo. afzelii* (2.8%, 30/1063); *Bo. garinii* (4.6%, 49/1063); *Bo. valaisiana* (3.9%, 42/1063); *Bo. burgdorferi* (0.2%, 2/1063); *Borrelia turdi* (0.2%, 2/1063); *Bo. lusitaniae* (0.1%, 1/1063); and *Bo. miyamotoi* (0.9%, 10/1063).

#### 2.1.4. Prevalence of *Babesia* Species in *I. ricinus* Removed from Birds 

In the ticks collected from birds at Ottenby in South-Eastern Sweden, totals of 514 larvae and 532 nymphs of *I. ricinus* were analysed for *Babesia* species, i.e., *Ba. microti, Ba. divergens*, *Ba. venatorum* and *Ba. capreoli* [42]. The overall prevalence of *Babesia* spp. In immature *I. ricinus* from birds was 2.5% (26/1046). Among these 26 immature ticks were 9 larvae and 6 nymphs positive for *Ba. venatorum*, 10 nymphs positive for *Ba. microti* and 1 larva positive for *Ba. capreoli* [42]. This supports the view that *Ba. venatorum* and *Ba. capreoli,* but not *Ba. microti*, are transovarially transmitted in *I. ricinus.*

### 2.2. Coinfection Analysis

#### 2.2.1. Coinfection of *Babesia* and *Borrelia* in *I. ricinus* Removed from Humans

Among ticks that were collected while attached to humans, 3.1% (60/1952) of nymphs and adult ticks were positive for possibly zoonotic *Babesia* spp. Of these *Babesia*-positive ticks, 43.3% (26/60) were concurrently *Borrelia*-positive. The prevalence of *Babesia* infections (incl. *Ba. capreoli*) in ticks derived from humans was twice as high (*p* < 0.01) in *Borrelia*-positive ticks (5.5%; 30/549) as in *Borrelia*-negative ticks (2.4%; 35/1472). Among the adult female and male ticks, 4.1% (21/511) were PCR-positive for *Babesia* spp. Among nymphs, 2.8% (43/1510) were *Babesia*-positive (Table 2). These proportions are not significantly different at *p* < 0.05. There were 13 adult ticks and 20 nymphs that were positive for *Ba. microti*; 7 adult ticks and 20 nymphs positive for *Ba. venatorum*; and 1 adult tick, 3 nymphs and 1 larva positive for *Ba. capreoli* (Table 2). Among 27 *Babesia*- and concurrently *Borrelia*-positive nymphs and adult ticks, most, i.e., 17 ticks (63%), were infected with *Ba. microti* and *Bo. afzelii* (Table 2). Five ticks (19%) were coinfected with *Ba. venatorum+Bo. afzelii*. The following coinfections were encountered once each: *Ba. microti*+*Bo. valaisiana* (one nymph); *Ba. microti+Bo. garinii* (one nymph); *Ba. venatorum+Bo. miyamotoi* (one adult female); *Ba. capreoli*+*Bo. burgdorferi* (one adult female); and *Ba. venatorum*+*Bo. garinii* (one nymph).

**Table 2:** Coinfection of *Borrelia* species in 65 *Babesia*-positive *Ixodes ricinus* ticks that had bitten people in Sweden or on the Åland Islands, Finland. Data from Table A1 in [85].

	*Bo. afzelii*	*Bo. garinii*	*Bo. valaisiana*	*Bo. burgdorferi*	*Bo. miyamotoi*	*Borrelia*-Negative	Not Performed
*Ba. microti* positive adult ticks (n = 13)	6	0	0	0	0	5	2
*Ba. venatorum* positive adult ticks (n = 7)	1	0	0	0	1	5	0
*Ba. capreoli* positive adult ticks (n = 1)	0	0	0	1	0	0	0
*Ba. microti* positive nymphs (n = 20)	11	1	1	0	0	7	0
*Ba. venatorum* positive nymphs (n = 20)	4	1	0	0	0	14	1
*Ba. capreoli* positive nymphs (n = 3)	0	0	0	0	0	3	0
*Ba. capreoli* positive larva (n = 1)	0	0	0	0	0	1	0

#### 2.2.2. Coinfection of *Babesia* and *Borrelia* in *I. ricinus* Removed from Birds 

The overall prevalence of possibly zoonotic *Babesia* spp. infections in immature ticks from birds was 2.5% (26/1046). The corresponding prevalence, 3.1%, in nymphal and adult ticks from humans was not significantly different at *p* < 0.05.

Ten of twenty-six (38.5%) *Babesia*-infected immature ticks removed from birds were concurrently infected with *Borrelia* (Table 3). The proportion of *Babesia*-positive among *Borrelia*-positive immature ticks, 3.51%, (10/285) was not significantly different (at *p* < 0.05) from that of *Babesia*-positive among *Borrelia*-negative immature ticks, 2.04% (16/783). The numbers of *Borrelia*-positive and *Borrelia*-negative immature *I. ricinus* ticks are provided in Table 2 in [103]. In total, 3 of 10 *Babesia*-positive larvae and 7 of 16 *Babesia*-positive nymphs were coinfected with *Borrelia* spp. (including 1 *Ba. microti*-infected nymph coinfected with an untypeable *Borrelia* sp., Table 3). Among these immature ticks removed from birds, there were three of each of the combinations *Ba. microti*+*Bo. afzelii* and *Ba. venatorum*+*Bo. valaisiana*; two of *Ba. venatorum+Bo. garinii*; and one of *Ba. venatorum+Bo. miyamotoi* (Table 3). Among coinfected immature ticks, three larvae and three nymphs were infected with *Ba. venatorum* and *Borrelia* species, the latter of which included *Bo. garinii*, *Bo. valaisiana* and *Bo. miyamotoi*.

## 3. Discussion

### 3.1. Coinfection of Babesia and Borrelia in Ticks Removed from Humans 

The most obvious explanation for particular combinations of pathogen coinfections is that nymphs acquired them all as larvae from the same reservoir host. However, there are several instances where this is questionable. Although three publications [39,40,41] suggest that birds can infect *Ixodes* larvae with *Ba. microti,* birds are generally not considered to be reservoir hosts of *Ba. microti* (page 3). Therefore, alternative possibilities will be considered for coinfections such as *Ba. microti*+*Bo. valaisiana* and *Ba. microti*+*Bo. garinii* in nymphs (Table 2), in which *Ba. microti* will have been acquired from small mammals and *Bo. valaisiana* and *Bo. garinii* from birds. In these cases, it is conjectured that either the larval blood meal was interrupted so that the larvae had fed on both a mammal and a bird or that cofeeding transmission had occurred, as discussed by Pichon et al. [106]. However, neither pathogen is known to be associated with this form of transmission. Experiments are necessary to prove (or disprove) if some bird species are competent reservoirs for *Ba. microti*. A nymph with the coinfection *Ba. venatorum*+*Bo. garinii* (Table 2) probably acquired the *Ba. venatorum* infection transovarially and the *Bo. garinii* infection from a bird. An adult female tick infected with either *Ba. venatorum* or *Ba. capreoli* (Table 2) will probably have contracted its *Babesia* infection transovarially. The adult females infected with *Bo. miyamotoi* or *Bo. burgdorferi* sensu lato (Table 2) could have contracted both bacteria from infective mammals or birds, with the strong additional possibility that *Bo. miyamotoi* was acquired transovarially [107,108].

### 3.2. Coinfection of Babesia and Borrelia in Ticks Removed from Birds 

The larva infected with *Ba. venatorum* and *Bo. miyamotoi* (Table 3) probably contracted both microorganisms transovarially. *Bo. garinii* and *Bo. valaisiana* are associated with avian reservoirs, while *Bo. miyamotoi* can use both avian and mammalian reservoirs [109,110]. As mentioned, the latter is also transovarially transmitted [109]. Three of the coinfected nymphs were infected with *Ba. microti* and *Bo. afzelii* (Table 3), both of which would presumably have been acquired from small mammals. 

### 3.3. Babesia and Borrelia Coinfection in Humans

The data show that possibly zoonotic *Babesia* protozoans are abundantly present as coinfections with human-pathogenic *Borrelia* spirochaetes in *I. ricinus* ticks in Sweden and on the Åland archipelago. Such coinfections are common both among ticks that bite people and among ticks that infest migratory and resident birds in Southern Sweden. In the United States, it has been shown that both infections can be transmitted simultaneously by *I. scapularis* ticks [111]. In our studies, the prevalence of *Babesia* infections in nymphs and adults of *I. ricinus* biting humans was twice as high in *Borrelia*-infected ticks (5.5%) as in *Borrelia*-negative ticks (2.4%) [85]. 

The risk of developing LB after a bite from a tick presumed to be infected with a single species of *Bo. burgdorferi* s.l. has been estimated to be 2.6% in the Netherlands, but the risk increases with the duration of an infected tick attachment [112]. In a similar study in Sweden, the risk of developing LB after a tick bite was estimated to be 2.1% and was also found to increase with the duration of tick feeding [113]. In the United States, coinfection with *Babesia* and *Borrelia* is generally considered to cause more severe disease symptoms than a monoinfection with either *Babesia* or *Borrelia* [2,95]. A coinfection may be suspected if a patient first diagnosed as having contracted LB (often with erythema migrans) has high fever for more than two days despite anti-*Borrelia* antibiotic treatment or exhibits night sweats, anaemia, leukopenia or thrombocytopenia [114]. If a coinfection with *Babesia* and *Borrelia* in Europe causes a more severe disease than a monoinfection with either *Babesia* or *Borrelia,* it would follow that, if the biting tick were coinfected, the risk of developing disease could be at least ~2–3%. However, it appears that few of the coinfecting *Ba. microti* genotypes in Europe are as virulent as in the United States, and there is so far no evidence of exacerbation by *Babesia* infections in Europe—possibly as a result of a lack of research.

In a study in the Netherlands, Jahfari et al. [81] found that the probability of infection with a tick-borne pathogen (TBP) other than LB spirochaetes after a tick bite is roughly 2.4%. Among patients with erythema migrans, the probability of a coinfection with another TBP is approximately 2.7%. But how often these coinfections cause more severe disease symptoms requires further investigations [81]. 

The public health impact and economic significance of tick-transmitted coinfections have been emphasised in the United States during the last few years. There, the Tick-Borne Disease Working Group (TBDWG) has the mandate to review federal activities and research on all tick-associated diseases and to provide recommendations to the HHS Secretary and Congress every two years [115,116]. Thereby, the capacity to detect and treat LB and tick-borne coinfections in the United States has improved drastically during the last few years. 

In Europe, physicians and biomedical laboratory staff should be aware of the tendency for *Bo. burgdorferi* s.l. and *Babesia* parasites to occur together in ticks feeding on people. This is relevant for the rapid, adequate and correct diagnosis and treatment of tick-transmitted diseases, particularly in the elderly. Yet, in Sweden, it appears that few, if any, hospitals and health centres have the capacity to diagnose babesiosis. One of the real-time PCR DNA tests recently introduced for the diagnosis of blood infected with *Ba. microti* in the United States [117,118,119,120] could possibly be useful for the diagnosis of infections due to *Ba. microti, Ba. divergens* and *Ba. venatorum* in Europe.

### 3.4. Are There Undetected Coinfections?

The possibility that an individual *I. ricinus* tick may be infected with more than one human pathogen is an unexplored, neglected public health issue in Sweden. People may contract a simultaneous infection with *Bo. burgdorferi* s.l. and a potentially human-pathogenic *Babesia* species that rarely causes any symptoms of disease, at least not in immunocompetent people. One possible reason for this “negligence” may be that many laboratory methods cannot detect certain TBPs if they transform and “hide” in a resting state, “hide” at very low or almost undetectably low concentrations, or resist treatment by being unavailable to antibiotic molecules by staying in inaccessible tissues or organs. Finally, there may still be species of pathogenic, tick-transmitted microbes that are not yet known to science. Increased investigations are required on the disease potential of coinfections of zoonotic species of *Babesia*, *Borrelia, Anaplasma*, *Rickettsia*, *Neoehrlichia*, TBE virus and potentially other microbial and viral species hitherto unknown, not yet investigated, or not yet regarded as human pathogens transmitted by *I. ricinus* [121,122].

In Northern Europe, Gyllemark and coworkers recently performed such investigations. They studied the possible presence and clinical impact of TBPs, other than *Bo. burgdorferi* s.l., in the cerebrospinal fluid (CSF) and serum samples from 600 patients from South-Eastern Sweden who were suspected of having Lyme neuroborreliosis (LNB) [123]. The samples were investigated for *Babesia* spp., *Bo. burgdorferi* s.l., *Bo. miyamotoi*, *A. phagocytophilum*, *Rickettsia* spp. *N. mikurensis* and TBEV [123]. Some patients with CSF pleocytosis had *Borrelia*-specific antibodies in the CSF. One patient was PCR-positive for *N. mikurensis* in serum, and another one was PCR-positive for *Borrelia* spp. in serum. A few patients’ sera were positive for antibodies to *Bo. miyamotoi, A. phagocytophilum*, *Rickettsia* spp. or TBE virus [123]. Nine sera had antibodies to more than one pathogen. These results suggest that in patients showing symptoms in South-Eastern Sweden, who are investigated for LNB, TBPs are not commonly detected [123]. The absence of *Babesia* spp., which parasitise erythrocytes, could be due to the fact that only serum and CSF samples and not whole-blood samples were investigated. Alternatively, people may rarely become infected with human-pathogenic *Babesia* s.s. parasites because of the relatively low infection prevalence of these pathogens in *I. ricinus* ticks. 

Azagi and co-researchers recently investigated information from 2008 to 2018 on *I. ricinus*-vectored microorganisms (except *Bo. burgdorferi* s.l. and TBE virus) in Europe with the aim of evaluating the evidence for a causal relationship between infection and disease [67]. These scientists concluded that the evidence for human disease causality was strongest for *A. phagocytophilum* and *Ba. divergens* and that comprehensive evidence is missing for the other *I. ricinus*-borne microorganisms [67].

### 3.5. Effects of Coinfection on the Epidemiology of Human Babesiosis

In our study on *I. ricinus* ticks removed from humans, *Borrelia*-positive ticks were more commonly infected with *Babesia* spp. than *Borrelia*-negative ticks. This agrees with other similar studies. For instance, Pawelczyk and colleagues, studying ticks removed from humans in Poland, found 2.7% *Babesia* infection prevalence in *Borrelia*-positive ticks as against 0.8% in ticks uninfected with *Borrelia* [65]. Similar to our data, the Polish study showed that the most frequent coinfections involve *Bo. afzelii* and *Ba. microti* [65]. The ticks carrying these coinfections have, most likely, been infected as larvae from small mammals. 

Our data show that the prevalence of *Bo. burgdorferi* s.l. in *I. ricinus* from both humans and birds is much higher than that of *Babesia* spp. This could be one of several reasons that explain the high incidence of human LB and the much lower incidence of human babesiosis. A review by Eisen [124] of laboratory experiments in the United States showed that by 48 h after the attachment of a single *I. scapularis* nymph infected with *Bo. burgdorferi* s.s., the probability of transmission resulting in host (mouse) infection was ~10%, increasing to 50% by 63–67 h. In contrast, when multiple infected nymphs fed together, the probability of transmission of *Bo. burgdorferi* was, on average, 68% by 48 h after attachment. In two similar studies on the duration of transmission of *Ba. microti* by multiple *I. scapularis* nymphs, the probability of experimental hosts becoming infected was 50–71% [124]. These experiments suggest that *Bo. burgdorferi* and *Ba. microti* are transmitted by infective nymphal ticks at a similar rate after having attached to their hosts. This does not explain the generally much lower prevalence of *Ba. microti* (0.2–57.1%) compared to that of *Bo. burgdorferi* (6.0–71.4%) in field-collected *I. scapularis* nymphs in the Northeast and Upper Midwest of the United States [124]. 

Hersh et al. [125] collected questing nymphs of *I. scapularis* and recorded 83% more coinfection with *Ba. microti* and *Bo. burgdorferi* than predicted by chance alone. This pattern of higher levels of coinfection was observed in tick larvae that had fed on small mammals but not in larvae fed on larger mammals or on birds [125]. The transmission of *Ba. microti* seems immunologically facilitated in rodents infected with an invasive strain of *Bo. burgdorferi* [3]. This may explain the increased coinfection rates with *Ba. microti* and *Bo. burgdorferi* observed in larval and nymphal *Ixodes* ticks that have fed on rodents [3,4,125]. Levels of coinfection rates of other tick-borne microorganisms with deviations from those expected by chance alone have been described by Kurtenbach et al. [126], Ginsberg [127], Herrmann et al. [128], Dunn et al. [3] and Cutler et al. [74]. The reason(s) that coinfections occurred more often than expected may be that the reservoirs, i.e., mainly small mammals, may have equal tolerance for both these pathogens, resulting in them assorting together rather than independently [125]. Another reason may be that one of the pathogens facilitates infection with or transmission of the other [125]. A third possibility is that certain individual hosts, particularly male rodents, are particularly prone to become infested by infected nymphs due to their high activity, and are also infested by many larval ticks, which could increase the prevalence of coinfected nymphs [125]. A fourth hypothesis is that one of the pathogens confers a survival advantage in the host to the other pathogen and vice versa. For example, Diuk-Wasser et al. showed that, in the United States, coinfection with *Bo. burgdorferi* increases the suitability of *P. leucopus* as a reservoir host for *Ba. microti* [4].

It has been recently estimated that the annual number of symptomatic LB cases now exceeds 450,000 in the United States [129]. However, it cannot be excluded that an unknown proportion of these cases might include other illnesses misdiagnosed as LB [130]. Yet, there are several reasons for the increasing number of estimated cases of LB recorded both in the United States and Europe: improved knowledge and heightened awareness, better diagnostic methods, an aging human population with a less effective immune defence, and most importantly, climate change and coincident drastic ecological changes in the environment. In the United States, the latter include reforestation and other changes in the vegetation that have favoured the population of white-tailed deer (*Odocoileus virginianus*), the main host of adult *I. scapularis,* the main vector of *Bo. burgdorferi* in that country. The increasing prevalence and geographic expansion of *Bo. burgdorferi* there have been followed by an increasing prevalence and geographic distribution of *Ba. microti* in the *Ixodes* and small mammal populations [3,4], and by increasing incidence of human babesiosis [4]. The reasons for the increasing number of human cases of babesiosis in the United States include enhanced public health awareness and the increased potential for *Ba. microti* to be transmitted and established in new areas where *Bo. burgdorferi*-infected ticks and small mammals are present. 

The incidence and geographic distribution of human babesiosis in the United States have increased considerably during the last few decades [5,6,89,95,131,132]. In areas of the United States, where both *Bo. burgdorferi* and *Ba. microti* are enzootic, about 11% of early LB patients have concurrent babesiosis, while, on average, 52% of patients with babesiosis have simultaneous LB [2,23,95,96]. In the North-Eastern United States, coinfection of *Ba. microti* and *Bo. burgdorferi* is prevalent, up to 13%, in *I. scapularis* [89] and also in one of the important small mammal reservoirs, the white-footed mouse, of both pathogens [41,92,125]. 

Ecological and epidemiological data suggest that the situation in Europe is different. An increasing incidence of human babesiosis, as in North America, has not been observed in Europe. Yet, the ecology and epidemiology of LB and some other tick-borne infections of humans in the two regions have certain features in common. Climate change, increasing abundance of deer, their gradually increasing synanthropy, and an aging human population are factors common to Northern Europe and North America. The roe deer is a reservoir in Sweden of *Ba. venatorum* and *Ba. capreoli* [36]. The Swedish populations of cervids, particularly the roe deer population, expanded rapidly during the late 1980s and early 1990s, which, together with a warmer climate, resulted in a dramatic increase in and range expansion of the population of the common tick, *I. ricinus.* In Sweden, this species is the main vector of *Bo. burgdorferi* s.l.; of three species of zoonotic *Babesia* species, i.e., *Ba. divergens*, *Ba. microti* and *Ba. venatorum*; and of some other human pathogens. The population of *I. ricinus* was estimated to have increased approximately tenfold from the early 1990s to 2009 in Southern Sweden when this tick also extended its area of distribution in Northern Sweden from about 13% to about 27% [16,17]. There are several changes in tick abundance also reported from other European countries. For instance, Bregnard et al. [133] showed that the abundance of *I. ricinus* at a location in Switzerland almost doubled from 2004 to 2018. There is also evidence for an increase in the total number of *I. ricinus* ticks in the Netherlands [12], in the United Kingdom [134] and in other European countries [15]. 

The increasing abundance of *I. ricinus* in many European regions has been accompanied by increasing numbers of cases of tick-borne diseases (TBDs) such as TBE and LB [135]. For instance, between 2005 and 2021, the annual number of serious cases of TBE in humans in Sweden increased from 131 cases to 512 [136,137]. The annual incidence of human LB has not been recorded for Sweden, but such data are available for neighbouring Finland. Here, the incidence of LB increased from 345 cases in 1995 to 2570 cases in 2021 [138]. Similarly, the annual incidence of TBE recorded in mainland Finland (excluding the Åland archipelago, where TBE vaccination has reduced the incidence drastically) was 3 cases in 1995 and increased to 127 in 2021 [138]. These data seem to lend strong supporting evidence to the notion that the human incidence of several TBDs has increased dramatically during this century in Northern Europe. Bajer and coworkers recently reviewed the epidemiology of babesiosis in humans and domesticated animals in 20 European countries [25]. They concluded that human cases are expected to rise due to increased abundance of *I. ricinus* as a result of climate change; bovine babesiosis has a re-emerging potential because of the likely loss of herd immunity; and canine babesiosis is rapidly expanding as a result of the increasing abundance and range of *D. reticulatus.* However, even if this is true, we cannot dismiss the possibility that one or more confounding factors cause the data to look as if the incidence of TBDs has increased more than it really has. In summary, the incidence of TBDs has in reality increased, but we need to accept that the numbers of human cases of TBE, LB and other TBDs recorded are at least partly influenced by confounding factors such as the increased attention given to TBDs and improved methods to detect them. 

The fact that in both the United States and Europe the seroprevalence of *Babesia* infections in humans far exceeds clinical incidence indicates that many people contract subclinical or asymptomatic infections [139,140,141,142]. Even in Sweden, serological information [102] and vector biological data on the *Babesia* spp. infection rates in *I. ricinus* [56] indicate that human cases of babesiosis might be more prevalent than indicated by the actual number of cases registered. In contrast to this, Nilsson and coworkers did not detect anti-*Babesia* IgG antibodies in any of their 224 individuals with chronic symptoms suspected to be (indirectly) caused by tick bites [143]. 

In fact, only two cases of human clinical babesiosis—caused by *Ba. divergens* and *Ba. venatorum*—have been reported in Sweden so far [144,145]. In neighbouring Norway, where in some areas as in Sweden, *Ba. divergens* occurs in cattle and *I. ricinus* [55], only one human case of *Ba. divergens* babesiosis is on record [146]. Thus, despite a relatively high seroprevalence in humans [102,147] and presence in *I. ricinus* [42,56,73,85,148,149] in Northern Europe, the risk that both healthy persons and immunocompromised persons will develop symptomatic babesiosis after a *Babesia* infection appears low [85]. However, the *Babesia* spp. seroprevalence in humans and infection prevalence in the vector population suggest that human babesiosis in Sweden and elsewhere in Europe could be an underdiagnosed infection [102,123,150]. The possibility that tick-borne and transfusion-transmitted zoonotic babesiosis is a more important medical problem than hitherto recognised has also been addressed elsewhere [102,115,151]. As remarked by Sam Telford III, “piroplasms should routinely be sought as an aetiology for febrile illnesses wherever humans are intensely exposed to ticks” [20].

### 3.6. Persistent LB-like Symptoms due to Chronic Babesiosis?

In a study conducted in the Netherlands [151], the prevalence of long-lasting symptoms and of severe symptoms were recorded during one year in the hitherto largest prospective group of adults with physician-confirmed LB and compared with two control cohorts; persistent symptoms were significantly more prevalent and more severe in LB patients than in individuals in the reference cohorts, which suggests an association between LB and persistent symptoms. Fatigue, cognitive impairment and pain were the most frequently reported persistent symptoms in the LB population [151].

As already emphasised, coinfections with *Babesia* spp. and *Borrelia* spp. are not rare in *Ixodes* ticks, in the small mammals that are reservoirs of the pathogens, and in humans, both in the United States and in Europe. Coinfection(s), such as babesiosis, remaining in some patients who have been treated for LB, may explain persistent symptoms in some patients [112,152,153]. A silent *Babesia* infection that is left untreated in immunocompetent persons sometimes disappears after a long time without any specific pharmacological treatment. In other persons, the infection may persist for months or even years [18,24,95,153]. Thus, persons infected with *Ba. microti* can become chronic, asymptomatic carriers, indicated by the many cases of transfusion-transmitted babesiosis that have occurred in the United States [18,115,154]. In such cases, diagnosis should not rely on serological tests; rather, it is advisable to detect the coinfecting pathogen by PCR, by microscopy combined with specific staining, or by xenodiagnosis using rodents. Molecular tools are required for species identification [24]. Attempts to demonstrate babesiae and other TBPs such as *Bo. miyamotoi* and *A. phagocytophilum* are, in most European clinical settings, exceptional rather than common practice. 

Guidelines for the prevention, diagnosis, and treatment of LB, although primarily focusing on North America, recommend that when patients present with a high-grade fever or characteristic laboratory abnormalities, clinicians should consider a possible coinfection with *Ba. microti* and/or *A. phagocytophilum*, particularly when the patient has visited a region where these pathogens are known to occur. Such coinfections should also be investigated in patients who have a persistent fever for >1 day while on antibiotic treatment for LB. If the fever persists despite treatment with doxycycline (the antibiotic of choice for *A. phagocytophilum* infection), a *Ba. microti* infection should be considered. Characteristic laboratory abnormalities found in both babesiosis and anaplasmosis include thrombocytopaenia, leukopaenia, neutropaenia, and/or anaemia. Evidence of haemolysis, such as elevated indirect bilirubin level, anaemia, and elevated lactate dehydrogenase, is particularly suggestive of babesiosis” [155].

The exacerbation of LB symptoms by concurrent *Ba. microti* infections [4,96,156] requires laboratory evaluation for both pathogens in LB patients with persistent symptoms [95,115]. Yet, laboratory tests for babesiosis are rarely performed on patients positive for LB or for those suspected of suffering from Post-Treatment Lyme Disease Syndrome (PTLDS). Some patients with persistent forms of LB, perhaps sometimes erroneously diagnosed as PTLDS cases, might possibly be infected simultaneously with *Bo. burgdorferi* s.l. and a zoonotic *Babesia* species. If such patients only receive antibiotics against the *Borrelia* infection, the *Babesia* infection may persist and could, if not adequately treated, later be misdiagnosed as PTLDS. Therefore, any LB patient who remains symptomatic despite adequate anti-*Borrelia* antibiotic treatment should preferably be tested early for the presence of other microorganisms [156], particularly babesiae. It should be noted that influenza-like symptoms, anaemia, thrombocytopenia and splenomegaly are uncommon symptoms in LB patients but can be indicators of a *Babesia* coinfection, particularly if adequate treatment with antibiotics recommended for LB does not resolve the illness [23,95].

A recent seroprevalence study investigated the possible contribution of *A. phagocytophilum*, *Bartonella henselae*/*quintana* and *Ba. microti* to the aetiology of PTLDS [152]: the authors detected a wide spectrum of autoantibodies in some of the patients with complicated types of LB and PTLDS but did not find a significant impact of coinfections in the patients investigated [152]. The causes of PTLDS are not yet fully understood, but some of the hypotheses for its aetiology are the remaining antigenic activity of nonviable spirochaetes, viable spirochaetes unavailable to antibiotic molecules, inappropriate immune activation, coinfection(s), or some of these factors in combination [157].

### 3.7. Babesia-Infected Transfusion Blood

Many tick-borne pathogens, such as *A. phagocytophilum* [158,159], *Ehrlichia chaffeensis* [158], *Ba. microti* [158,160], *Ba. divergens*, *Ba. venatorum* [158] and *Bo. miyamotoi* [161], can be transmitted by blood transfusion [162,163,164,165]. Most cases describing patients infected with TBPs via blood transfusion or organ transplants have occurred in the United States, where transfusion-transmitted babesiosis has caused severe complications and death in about 20% of cases [154]. It is likely that the risk to European patients is much lower due to the generally lower virulence of European strains of *Ba. microti* and the general lack of persistence in patients of some others such as *Ba. divergens.* The potential risk to immunocompromised patients receiving TBP-infected transfusion blood, donated by immunocompetent TBP-infected and asymptomatic donors, should, however, not be dismissed [165,166]. Blood from such silent carriers is sometimes used—unintentionally—for transfusions. Healthy individuals who have become infected by tick-borne babesiae are usually asymptomatic or present with a relatively benign, mild, influenza-like illness [24,95,120,162,163]. In contrast, splenectomised or otherwise immunocompromised individuals who receive *Babesia*-infected or *Anaplasma*-infected blood transfusions are much more likely to develop severe disease [120,166,167,168]. In view of the potentially high mortality rate among immunodeficient patients receiving such infected blood, its usage for transfusions must be avoided [166]. The mortality rate, about 20%, among this group of immunodeficient patients is similar to that among such patients who acquire the infection from an infective tick [95]. In view of the fact that asymptomatic carriers of babesiae exist and that such individuals may donate blood, TBPs are a real threat to the blood supply in the United States and presumably also in Europe. Screening for pathogens in donated blood and blood products has recently been implemented in the United States [118,119,120,166,167] and seems necessary also in Europe. Infections with *Ba. microti* due to blood transfusion in immunocompromised persons are easily missed since most of these patients do not develop symptoms immediately after transfusion, but usually after a relatively long incubation period (1–9 weeks up to 6 months) [95,120,166]. There are now several methods available for reducing or eliminating different pathogens, including babesiae, in blood and blood components intended for transfusion [166]. Yet, tests for the routine detection of TBPs in blood therapy are not implemented in most European hospitals.

## 4. Conclusions

To be able to correctly diagnose a TBD, one needs to take into account potential coinfection(s). This is necessary to select the correct treatment and because of the possibility that a coinfection may aggravate the illness. Babesiosis should be suspected in any febrile, nonspecific syndrome where at least a potential exposure to tick bite can be suspected. The relatively high *Babesia*+*Borrelia* coinfection prevalences in *I. ricinus*, a tick which is a ubiquitous blood-feeding ectoparasite on humans, suggest that coinfections with zoonotic species of *Babesia* and *Borrelia* may be a hitherto-neglected but potentially important public health issue in Northern Europe. In view of the often-nonspecific symptoms and potential severity of human babesiosis in the older population, increased alertness to this infection is recommended. It should be obvious that the best prophylaxis against TBPs is to avoid tick-infested habitats. People who nevertheless visit such habitats should wear boots and suitable clothing. If attached ticks are found, they should be removed as soon as possible.

**Future research needs**

- Whether subclinical, mild babesiosis and transfusion-transmitted TBPs are significant medical problems in Northern Europe should be investigated.
- Research on the identification, characterization and phylogenetics of *Ba. microti*, *Ba. divergens*-like parasites and other *Babesia* species with putative zoonotic potential should be strengthened to understand their medical importance.
- Investigations are required concerning the vector competence of different *Ixodes* spp., particularly *I. persulcatus* and *I. trianguliceps,* to transmit different genotypes/strains of *Ba. microti*, and the vector biology of *I. ricinus* related to the Munich strain of *Ba. microti.*
- Investigations regarding the potential of birds to serve as reservoirs for different genotypes of *Ba. microti* are warranted in view of earlier indications that birds may be reservoirs for *Ba. microti* or related parasites [39,40,41].

## Figures and Tables

**Table 1:** Prevalence (%) of *Babesia* spp. in *Ixodes ricinus* larvae, nymphs, and/or adults in Northern Europe.

*Ba. microti* Clade 1	*Ba. divergens* Clade X	*Ba. venatorum* Clade X	*Ba. capreoli* Clade X	*Babesia* sp. *	Tot. No. Ticks	Country	Sampling Method	Reference
	0.9				224	Norway	Cloth drag	[54]
	0.1	0.6	0.1	0.1	1908	Norway	Cloth drag	[55]
3.2	0.2	1			519	Sweden	Cloth drag	[56]
	2.1				140	Lithuania	Cloth drag	[54]
2.4		1.6		5.4	370	Lithuania	Cloth drag	[57]
0.7		0.7			432	Latvia	Cloth drag	[44]
	0.1	0.3			2014	Finland	Citizens were asked to provide ticks	[58]
		1.2			515	Finland	Cloth drag	[59]
3.3		0.6	0.2		539	C. Germany	Cloth drag	[60]
0.8		2.1			1276–1665	Estonia	Cloth drag	[61]
		0.9			540	Denmark	Cloth drag	[62]
13.0					514	Poland	Cloth drag	[63]
0.7		0.2		0.7	3165	Poland	Cloth drag	[64]
0.8		0.4		0.08	1115	Poland	Removed from humans	[65]
0.1	2.0				1328	Poland	Cloth drag	[66]
	0.01	0.8 (1.9, all Clade X)	0.04		25,849	Netherlands	Cloth drag	[67]
2.6					18,626	Netherlands	Cloth drag	[67]

* including *Ba. canis* and unidentified species of *Babesia*.

**Table 3:** Coinfection of *Babesia* and *Borrelia* in immature *Ixodes ricinus* ticks removed from birds at Ottenby Bird Observatory, South-Eastern Sweden.

Tick Developmental Stage	*Babesia* Species ^a^	*Borrelia* Species ^b^	Bird Species ^c^	Collection Period ^d^
Larva	*Ba. venatorum*	*Bo. miyamotoi*	*Troglodytes troglodytes*	Spring
Larva	*Ba. venatorum*	*Bo. garinii*	*Turdus merula*	Autumn
Larva	*Ba. venatorum*	*Bo. valaisiana*	*Troglodytes troglodytes*	Autumn
Nymph	*Ba. venatorum*	*Bo. valaisiana*	*Turdus merula*	Spring
Nymph	*Ba. venatorum*	*Bo. valaisiana*	*Erithacus rubecula*	Spring
Nymph	*Ba. venatorum*	*Bo. garinii*	*Turdus merula*	Autumn
Nymph	*Ba. microti*	*Bo. afzelii*	*Turdus merula*	Autumn
Nymph	*Ba. microti*	*Bo. afzelii*	*Anthus trivialis*	Spring
Nymph	*Ba. microti*	*Bo. afzelii*	*Phoenicurus phoenicurus*	Spring
Nymph	*Ba. microti*	untypeable	*Turdus merula*	Spring

^a^ *Babesia* species detected previously in [42]. ^b^ *Borrelia* species detected previously in [103]. ^c^ bird species on which *I. ricinus* was found. ^d^ ticks were collected from the birds during 15 March–15 June and 15 July–15 November 2009.

## Data Availability

The data supporting the conclusions of this article are included within the article. Raw data can be shared with researchers upon a specific request.

## References

[1] Telfer S., Lambin X., Birtles R., Beldomenico P., Burthe S., Paterson S., Begon M. (2010). Species interactions in a parasite community drive infection risk in a wildlife population. Science.

[2] Krause P.J., Telford S.R., Spielman A., Sikand V., Ryan R., Christianson D. (1996). Concurrent Lyme disease and babesiosis: Evidence for increased severity and duration of illness. JAMA.

[3] Dunn J.M., Krause P.J., Davis S., Vannier E.G., Fitzpatrick M.C., Rollend L., Belperron A.A., States S.L., Stacey A., Bockenstedt L.K. (2014). *Borrelia burgdorferi* promotes the establishment of *Babesia microti* in the North-Eastern United States. PLoS ONE.

[4] Diuk-Wasser M.A., Vannier E., Krause P.J. (2016). Coinfection by *Ixodes* tick-borne pathogens: Ecological, epidemiological, and clinical consequences. Trends Parasitol..

[5] CDC—Centers for Disease Control (2019). CDC-Babesiosis—Disease. https://www.cdc.gov/parasites/babesiosis/disease.html.

[6] Ingram D., Crook T. (2020). Rise in babesiosis cases, Pennsylvania, USA, 2005–2018. Emerg. Infect. Dis..

[7] CDC (2019). Centers for Disease Control and Prevention. Lyme Disease Data and Surveillance. https://www.cdc.gov/lyme/datasurveillance/index.html.

[8] CDC (2018). Centers for Disease Control and Prevention. How Many People Get Lyme Disease?. https://www.cdc.gov/lyme/stats/humancases.html.

[9] Marques A.R., Strle F., Wormser G.P. (2021). Comparison of Lyme disease in the United States and Europe. Emerg. Infect. Dis..

[10] Sprong H., Azagi T., Hoornstra D., Nijhof A.M., Knorr S., Baarsma M.E., Hovius J.W. (2018). Control of Lyme borreliosis and other *Ixodes ricinus*-borne diseases. Parasites Vectors.

[11] Jahfari S., Fonville M., Hengeveld P., Reusken C., Scholte E.J., Takken W., Heyman P., Medlock J.M., Heylen D., Kleve J. (2012). Prevalence of *Neoehrlichia mikurensis* in ticks and rodents from North-West Europe. Parasites Vectors.

[12] Sprong H., Hofhuis A., Gassner F., Takken W., Jacobs F., van Vliet A.J., van Ballegooijen M., Takumi K. (2012). Circumstantial evidence for an increase in the total number and activity of *Borrelia*-infected *Ixodes ricinus* in the Netherlands. Parasites Vectors.

[13] Hofhuis A., Harms M., van den Wijngaard C., Sprong H., van Pelt W. (2015). Continuing increase of tick bites and Lyme disease between 1994 and 2009. Ticks Tick Borne Dis..

[14] Medlock J.M., Hansford K.M., Bormane A., Derdakova M., Estrada-Peña A., George J.C., Golovljova I., Jaenson T.G.T., Jensen J.-K., Jensen P.M. (2013). Driving forces for changes in geographical distribution of *Ixodes ricinus* ticks in Europe. Parasites Vectors.

[15] Jaenson T.G.T., Petersson E.H., Jaenson D.G.E., Kindberg J., Pettersson J.H., Hjertqvist M., Medlock J.M., Bengtsson H. (2018). The importance of wildlife in the ecology and epidemiology of the TBE virus in Sweden: Incidence of human TBE correlates with abundance of deer and hares. Parasites Vectors.

[16] Jaenson T.G., Hjertqvist M., Bergstrom T., Lundkvist A. (2012). Why is tick-borne encephalitis increasing? A review of the key factors causing the increasing incidence of human TBE in Sweden. Parasites Vectors.

[17] Jaenson T.G., Jaenson D.G., Eisen L., Petersson E., Lindgren E. (2012). Changes in the geographical distribution and abundance of the tick *Ixodes ricinus* during the past 30 years in Sweden. Parasites Vectors.

[18] Homer M.J., Aguilar-Delfin I., Telford S.R., Krause P.J., Persing D.H. (2000). Babesiosis. Clin. Microbiol. Rev..

[19] Telford S.R., Gorenflot A., Brasseur P., Spielman A., Kreier J.P. (1993). Babesial infections in humans and wildlife. Parasitic Protozoa.

[20] Telford S.R., Goethert H.K. (2004). Emerging tick-borne infections: Rediscovered and better characterized, or truly ‘new’?. Parasitology.

[21] Peirce M.A. (2000). A taxonomic review of avian piroplasms of the genus *Babesia* Starcovici, 1893 (Apicomplexa: Piroplasmorida: Babesiidae). J. Nat. Hist..

[22] Yabsley M.J., Vanstreels R.E.T., Shock B.C., Purdee M., Horne E.C., Peirce M.A., Parsons N.J. (2017). Molecular characterization of *Babesia peircei* and *Babesia ugwidiensis* provides insight into the evolution and host specificity of avian piroplasmids. Int. J. Parasitol. Parasites Wildl..

[23] Krause P.J. (2019). Human babesiosis. Int. J. Parasitol..

[24] Hildebrandt A., Zintl A., Montero E., Hunfeld K.P., Gray J. (2021). Human babesiosis in Europe. Pathogens.

[25] Bajer A., Beck A., Beck R., Behnke J.M., Dwużnik-Szarek D., Eichenberger R.M., Farkas R., Fuehrer H.-P., Heddergott M., Jokelainen P. (2022). Babesiosis in Southeastern, Central and Northeastern Europe: An emerging and re-emerging tick-borne disease of humans and animals. Microorganisms.

[26] Gray J.S., Estrada-Peña A., Zintl A. (2019). Vectors of babesiosis. Annu. Rev. Entomol..

[27] Bonnet S., Jouglin M., Malandrin L., Becker C., Agoulon A., L’hostis M., Chauvin A. (2007). Transstadial and transovarial persistence of *Babesia divergens* DNA in *Ixodes ricinus* ticks fed on infected blood in a new skin-feeding technique. Parasitology.

[28] Goethert H.K. (2021). What *Babesia microti* is now. Pathogens.

[29] Siński E., Bajer A., Welc R., Pawełczyk A., Ogrzewalska M., Behnke J.M. (2006). *Babesia microti*: Prevalence in wild rodents and *Ixodes ricinus* ticks from the Mazury Lakes District of North-Eastern Poland. Int. J. Med. Microbiol..

[30] Moniuszko-Malinowska A., Swiecicka I., Dunaj J., Zajkowska J., Czupryna P., Zambrowski G., Chmielewska-Badora J., Żukiewicz-Sobczak W., Swierzbinska R., Rutkowski K. (2016). Infection with *Babesia microti* in humans with non-specific symptoms in North East Poland. Infect. Dis. Lond. Engl..

[31] Arsuaga M., Gonzalez L.M., Lobo C.A., de la Calle F., Bautista J.M., Azcárate I.G., Puente S., Montero E. (2016). First report of *Babesia microti*-caused babesiosis in Spain. Vector Borne Zoonotic Dis..

[32] Bonnet S., Jouglin M., L’Hostis M., Chauvin A. (2007). *Babesia* sp. EU1 from roe deer and transmission within *Ixodes ricinus*. Emerg. Infect. Dis..

[33] Michel A.O., Mathis A., Ryser-Degiorgis M.P. (2014). *Babesia* spp. in European wild ruminant species: Parasite diversity and risk factors for infection. Vet. Res..

[34] Malmsten J., Dalin A.M., Moutailler S., Devillers E., Gondard M., Felton A. (2019). Vector-borne zoonotic pathogens in Eurasian Moose (*Alces alces alces*). Vector Borne Zoonotic Dis..

[35] Fanelli A. (2021). A historical review of *Babesia* spp. associated with deer in Europe: *Babesia divergens*/*Babesia divergens*-like, *Babesia capreoli*, *Babesia venatorum*, *Babesia* cf. odocoilei. Vet. Parasitol..

[36] Andersson M.O., Bergvall U.A., Chirico J., Christensson M., Lindgren P.E., Nordström J., Kjellander P. (2016). Molecular detection of *Babesia capreoli* and *Babesia venatorum* in wild Swedish roe deer, *Capreolus capreolus*. Parasites Vectors.

[37] Razanske I., Rosef O., Radzijevskaja J., Bratchikov M., Griciuviene L., Paulauskas A. (2019). Prevalence and co-infection with tick-borne *Anaplasma phagocytophilum* and *Babesia* spp. in red deer (*Cervus elaphus*) and roe deer (*Capreolus capreolus*) in Southern Norway. Int. J. Parasitol. Parasites Wildl..

[38] Gray J., Zintl A., Hildebrandt A., Hunfeld K.P., Weiss L. (2010). Zoonotic babesiosis: Overview of the disease and novel aspects of pathogen identity. Ticks Tick-Borne Dis..

[39] Franke J., Fritzsch J., Tomaso H., Straube E., Dorn W., Hildebrandt A. (2010). coexistence of pathogens in host-seeking and feeding ticks within a single natural habitat in central Germany. Appl. Environ. Microbiol..

[40] Hildebrandt A., Franke J., Meier F., Sachse S., Dorn W., Straube E. (2010). The potential role of migratory birds in transmission cycles of *Babesia* spp., *Anaplasma phagocytophilum*, and *Rickettsia* spp.. Ticks Tick-Borne Dis..

[41] Hersh M.H., Tibbetts M., Strauss M., Ostfeld R.S., Keesing F. (2012). Reservoir competence of wildlife host species for *Babesia microti*. Emerg. Infect. Dis..

[42] Wilhelmsson P., Pawełczyk O., Jaenson T.G.T., Waldenström J., Olsen B., Forsberg P., Lindgren P.E. (2021). Three *Babesia* species in *Ixodes ricinus* ticks from migratory birds in Sweden. Parasites Vectors.

[43] Gray J., von Stedingk L.V., Gürtelschmid M., Granström M. (2002). Transmission studies of *Babesia microti* in *Ixodes ricinus* ticks and gerbils. J. Clin. Microbiol..

[44] Capligina V., Berzina I., Bormane A., Salmane I., Vilks K., Kazarina A., Bandere D., Baumanis V., Ranka R. (2016). Prevalence and phylogenetic analysis of *Babesia* spp. in *Ixodes ricinus* and *Ixodes persulcatus* ticks in Latvia. Exp. Appl. Acarol..

[45] Rar V.A., Epikhina T.I., Livanova N.N., Panov V.V. (2011). Genetic diversity of *Babesia* in *Ixodes persulcatus* and small mammals from North Ural and West Siberia, Russia. Parasitology.

[46] Korenberg E.I., Nefedova V.V., Kovalevsky Y.V., Sorokina Y.V., Gorelova N.B. (2015). Parasitological factors impeding the transmission of the agent of babesiosis (*Babesia microti*) to man from the tick *Ixodes persulcatus*. Parazitologiia.

[47] Zamoto-Niikura A., Tsuji M., Qiang W., Nakao M., Hirata H., Ishihara C. (2012). Detection of two zoonotic *Babesia microti* lineages, the Hobetsu and U.S. lineages, in two sympatric tick species, *Ixodes ovatus* and *Ixodes persulcatus*, respectively, in Japan. Appl. Environ. Microbiol..

[48] Zamoto-Niikura A., Morikawa S., Hanaki K.I., Holman P.J., Ishihara C. (2016). *Ixodes persulcatus* ticks as vectors for the *Babesia microti* U.S. lineage in Japan. Appl. Environ. Microbiol..

[49] Sun Y., Liu G., Yang L., Xu R., Cao W. (2008). *Babesia microti*-like rodent parasites isolated from *Ixodes persulcatus* (Acari: Ixodidae) in Heilongjiang Province, China. Vet. Parasitol..

[50] Randolph S.E. (1995). Quantifying parameters in the transmission of *Babesia microti* by the tick *Ixodes trianguliceps* amongst voles (*Clethrionomys glareolus*). Parasitology.

[51] Randolph S.E. (1991). The effect of *Babesia microti* on feeding and survival in its tick vector, *Ixodes trianguliceps*. Parasitology.

[52] Krampitz H.E. (1979). Babesia microti: Morphology, Distribution and Host Relationship in Germany. https://www.cabi.org/ISC/abstract/19800866362.

[53] Bown K.J., Lambin X., Telford G.R., Ogden N.H., Telfer S., Woldehiwet Z., Birtles R.J. (2008). Relative importance of *Ixodes ricinus* and *Ixodes trianguliceps* as vectors for *Anaplasma phagocytophilum* and *Babesia microti* in field vole (*Microtus agrestis*) populations. Appl. Environ. Microbiol..

[54] Radzijevskaja J., Paulauskas A., Rosef O. (2008). Prevalence of *Anaplasma phagocytophilum* and *Babesia divergens* in *Ixodes ricinus* ticks from Lithuania and Norway. Int. J. Med. Microbiol..

[55] Oines O., Radzijevskaja J., Paulauskas A., Rosef O. (2012). Prevalence and diversity of *Babesia* spp. in questing Ixodes ricinus ticks from Norway. Parasites Vectors.

[56] Karlsson M.E., Andersson M.O. (2016). *Babesia* species in questing *Ixodes ricinus*, Sweden. Ticks Tick-Borne Dis..

[57] Radzijevskaja J., Mardosaitė-Busaitienė D., Aleksandravičienė A., Paulauskas A. (2018). Investigation of *Babesia* spp. in sympatric populations of *Dermacentor reticulatus* and *Ixodes ricinus* ticks in Lithuania and Latvia. Ticks Tick-Borne Dis..

[58] Laaksonen M., Klemola T., Feuth E., Sormunen J.J., Puisto A., Mäkelä S., Penttinen R., Ruohomäki K., Hänninen J., Sääksjärvi I.E. (2018). Tick-borne pathogens in Finland: Comparison of *Ixodes ricinus* and *I. persulcatus* in sympatric and parapatric areas. Parasites Vectors.

[59] Sormunen J.J., Andersson T., Aspi J., Bäck J., Cederberg T., Haavisto N., Halonen H., Hänninen J., Inkinen J., Kulha N. (2020). Monitoring of ticks and tick-borne pathogens through a nationwide research station network in Finland. Ticks Tick-Borne Dis..

[60] Silaghi C., Woll D., Hamel D., Pfister K., Mahling M., Pfeffer M. (2012). *Babesia* spp. and *Anaplasma phagocytophilum* in questing ticks, ticks parasitizing rodents and the parasitized rodents—Analyzing the host-pathogen-vector interface in a metropolitan area. Parasites Vectors.

[61] Katargina O., Geller J., Vasilenko V., Kuznetsova T., Järvekülg L., Vene S., Lundkvist Å., Golovljova I., Hamšíková Z., Kazimírová M. (2011). Detection and characterization of *Babesia* species in *Ixodes* ticks in Estonia. Vector Borne Zoonotic Dis..

[62] Klitgaard K., Kjær L.J., Isbrand A., Hansen M.F., Bødker R. (2019). Multiple infections in questing nymphs and adult female *Ixodes ricinus* ticks collected in a recreational forest in Denmark. Ticks Tick-Borne Dis..

[63] Skotarczak B., Rymaszewska A., Wodecka B., Sawczuk M. (2003). Molecular evidence of coinfection of *Borrelia burgdorferi* sensu lato, human granulocytic ehrlichiosis agent, and *Babesia microti* in ticks from North-Western Poland. J. Parasitol..

[64] Welc-Faleciak R., Bajer A., Paziewska-Harris A., Baumann-Popczyk A., Sinski E. (2012). Diversity of *Babesia* in *Ixodes ricinus* ticks in Poland. Adv. Med. Sci..

[65] Pawełczyk A., Bednarska M., Hamera A., Religa E., Poryszewska M., Mierzejewska E.J., Welc-Falęciak R. (2021). Long-term study of *Borrelia* and *Babesia* prevalence and co-infection in *Ixodes ricinus* and *Dermacentor recticulatus* ticks removed from humans in Poland, 2016–2019. Parasit Vectors.

[66] Pieniazek N., Sawczuk M., Skotarczak B. (2006). Molecular identification of *Babesia* parasites isolated from *Ixodes ricinus* ticks collected in northwestern Poland. J. Parasitol..

[67] Azagi T., Jaarsma R.I., Docters van Leeuwen A., Fonville M., Maas M., Franssen F.F.J., Kik M., Rijks J.M., Montizaan M.G., Groenevelt M. (2021). Circulation of *Babesia* species and their exposure to humans through *Ixodes ricinus*. Pathogens.

[68] Moutailler S., Valiente Moro C., Vaumourin E., Michelet L., Tran F.H., Devillers E., Cosson J.F., Gasqui P., Van V.T., Mavingui P. (2016). Co-infection of ticks: The rule rather than the exception. PLoS Neglected Trop. Dis..

[69] Raileanu C., Moutailler S., Pavel I., Porea D., Mihalca A.D., Savuta G., Vayssier-Taussat M. (2017). *Borrelia* diversity and co-infection with other tick borne pathogens in ticks. Front. Cell. Infect. Microbiol..

[70] Michelet L., Delannoy S., Devillers E., Umhang G., Aspan A., Juremalm M., Chirico J., van der Wal F.J., Sprong H., Pihl T.P.B. (2014). High-throughput screening of tick-borne pathogens in Europe. Front. Cell. Infect. Microbiol..

[71] Bonnet S., Michelet L., Moutailler S., Cheval J., Hébert C., Vayssier-Taussat M., Eloit M. (2014). Identification of parasitic communities within European ticks using next-generation sequencing. PLoS Neglected Trop. Dis..

[72] Nordberg M., Forsberg P., Berglund J., Bjöersdorff A., Ernerudh J., Garpmo U., Haglund M., Nilsson K., Eliasson I. (2014). Aetiology of tick-borne infections in an adult Swedish population—Are co-infections with multiple agents common?. Open J. Clin. Diagn..

[73] Capligina V., Seleznova M., Akopjana S., Freimane L., Lazovska M., Krumins R., Kivrane A., Namina A., Aleinikova D., Kimsis J. (2020). Large-scale countrywide screening for tick-borne pathogens in field-collected ticks in Latvia during 2017–2019. Parasit Vectors.

[74] Cutler S.J., Vayssier-Taussat M., Estrada-Peña A., Potkonjak A., Mihalca A.D., Zeller H. (2021). Tick-borne diseases and co-infection: Current considerations. Ticks Tick-Borne Dis..

[75] Kernif T., Leulmi H., Raoult D., Parola P. (2016). Emerging tick-borne bacterial pathogens. Microbiol. Spectr..

[76] Borşan S.D., Ionică A.M., Galon C., Toma-Naic A., Peştean C., Sándor A.D., Moutailler S., Mihalca A.D. (2021). High diversity, prevalence, and co-infection rates of tick-borne pathogens in ticks and wildlife hosts in an urban area in Romania. Front. Microbiol..

[77] Subramanian G., Sekeyova Z., Raoult D., Mediannikov O. (2012). Multiple tick-associated bacteria in *Ixodes ricinus* from Slovakia. Ticks Tick-Borne Dis..

[78] Gray J., Kahl O., Zintl A. (2021). What do we still need to know about *Ixodes ricinus*?. Ticks Tick-Borne Dis..

[79] Schulze T.L., Jordan R.A., Healy S.P., Roegner V.E. (2013). Detection of *Babesia microti* and *Borrelia burgdorferi* in host-seeking *Ixodes scapularis* (Acari: Ixodidae) in Monmouth County, New Jersey. J. Med. Entomol..

[80] Asman M., Witecka J., Korbecki J., Solarz K. (2021). The potential risk of exposure to *Borrelia garinii, Anaplasma phagocytophilum* and *Babesia microti* in the Wolinski National Park (north-western Poland). Sci. Rep..

[81] Jahfari S., Hofhuis A., Fonville M., van der Giessen J., van Pelt W., Sprong H. (2016). Molecular detection of tick-borne pathogens in humans with tick bites and erythema migrans, in the Netherlands. PLoS Neglected Trop. Dis..

[82] Henningsson A.J., Wilhelmsson P., Gyllemark P., Kozak M., Matussek A., Nyman D., Ekerfelt C., Lindgren P.-E., Forsberg P. (2015). Low risk of seroconversion or clinical disease in humans after a bite by an *Anaplasma phagocytophilum*-infected tick. Ticks Tick-Borne Dis..

[83] Richter D., Schlee D.B., Allgöwer R., Matuschka F.R. (2004). Relationships of a novel Lyme disease spirochete, *Borrelia spielmani* sp. nov., with its hosts in Central Europe. Appl. Environ. Microbiol..

[84] Stanek G., Strle F. (2018). Lyme borreliosis—From tick bite to diagnosis and treatment. FEMS Microbiol. Rev..

[85] Wilhelmsson P., Lövmar M., Krogfelt K.A., Nielsen H.V., Forsberg P., Lindgren P.E. (2020). Clinical/serological outcome in humans bitten by *Babesia* species positive *Ixodes ricinus* ticks in Sweden and on the Åland Islands. Ticks Tick-Borne Dis..

[86] Lommano E., Bertaiola L., Dupasquier C., Gern L. (2012). Infections and coinfections of questing *Ixodes ricinus* ticks by emerging zoonotic pathogens in Western Switzerland. Appl. Environ. Microbiol..

[87] Alekseev A.N., Semenov A.V., Dubinina H.V. (2003). Evidence of *Babesia microti* infection in multi-infected *Ixodes persulcatus* ticks in Russia. Exp. Appl. Acarol..

[88] Stewart P.E., Bloom M.E. (2020). Sharing the ride: *Ixodes scapularis* symbionts and their interactions. Front. Cell. Infect. Microbiol..

[89] Parveen N., Bhanot P. (2019). *Babesia microti*-*Borrelia burgdorferi* coinfection. Pathog. Basel Switz..

[90] Swanson S.J., Neitzel D., Reed K.D., Belongia E.A. (2006). Coinfections acquired from *Ixodes* ticks. Clin. Microbiol. Rev..

[91] Hofmeister E.K., Kolbert C.P., Abdulkarim A.S., Magera J.M., Hopkins M.K., Uhl J.R., Ambyaye A., Telford S.R., Cockerill F.R., Persing D.H. (1998). Cosegregation of a novel *Bartonella* species with *Borrelia burgdorferi* and *Babesia microti* in *Peromyscus leucopus*. J. Infect. Dis..

[92] Anderson J.F., Johnson R.C., Magnarelli L.A., Hyde F.W., Myers J.E. (1986). *Peromyscus leucopus* and *Microtus pennsylvanicus* simultaneously infected with *Borrelia burgdorferi* and *Babesia microti*. J. Clin. Microbiol..

[93] Benach J.L., Coleman J.L., Habicht G.S., MacDonald A., Grunwaldt E., Giron J.A. (1985). Serological evidence for simultaneous occurrences of Lyme disease and babesiosis. J. Infect. Dis..

[94] Grunwaldt E., Barbour A.G., Benach J.L. (1983). Simultaneous occurrence of babesiosis and Lyme disease. N. Engl. J. Med..

[95] Krause P.J., Auwaerter P.G., Bannuru R.R., Branda J.A., Falck-Ytter Y.T., Lantos P.M., Lavergne V., Meissner H.C., Osani M.C., Rips J.G. (2020). Clinical practice guidelines by the Infectious Diseases Society of America (IDSA): 2020 Guideline on Diagnosis and Management of Babesiosis. Clin. Infect. Dis..

[96] Sweeney C.J., Ghassemi M., Agger W.A., Persing D.H. (1998). Coinfection with *Babesia microti* and *Borrelia burgdorferi* in a western Wisconsin resident. Mayo Clinic Proceedings.

[97] Djokic V., Akoolo L., Primus S., Schlachter S., Kelly K., Bhanot P., Parveen N. (2019). Protozoan parasite *Babesia microti* subverts adaptive immunity and enhances Lyme disease severity. Front. Microbiol..

[98] Bhanot P., Parveen N. (2019). Investigating disease severity in an animal model of concurrent babesiosis and Lyme disease. Int. J. Parasitol..

[99] Hermanowska-Szpakowicz T., Skotarczak B., Kondrusik M., Rymaszewska A., Sawczuk M., Maciejewska A., Adamska M., Pancewicz S., Zajkowska J. (2004). Detecting DNA s of *Anaplasma phagocytophilum* and *Babesia* in the blood of patients suspected of Lyme disease. Ann. Agric. Environ. Med. AAEM.

[100] Arnez M., Luznik-Bufon T., Avsic-Zupanc T., Ruzic-Sabljic E., Petrovec M., Lotric-Furlan S., Strle F. (2003). Causes of febrile illnesses after a tick bite in Slovenian children. Pediatr. Infect. Dis. J..

[101] Haapasalo K., Suomalainen P., Sukura A., Siikamäki H., Jokiranta T.S. (2010). Fatal babesiosis in man, Finland, 2004. Emerg. Infect. Dis..

[102] Svensson J., Hunfeld K.P., Persson K.E. (2019). High seroprevalence of *Babesia* antibodies among *Borrelia burgdorferi*-infected humans in Sweden. Ticks Tick-Borne Dis..

[103] Wilhelmsson P., Jaenson T.G.T., Olsen B., Waldenström J., Lindgren P.E. (2020). Migratory birds as disseminators of ticks and the tick-borne pathogens *Borrelia* bacteria and tick-borne encephalitis (TBE) virus: A seasonal study at Ottenby Bird Observatory in South-Eastern Sweden. Parasites Vectors.

[104] Wilhelmsson P., Lindblom P., Fryland L., Ernerudh J., Forsberg P., Lindgren P.E. (2013). Prevalence, diversity, and load of *Borrelia* species in ticks that have fed on humans in regions of Sweden and Åland Islands, Finland with different Lyme borreliosis incidences. PLoS ONE.

[105] Wilhelmsson P., Fryland L., Börjesson S., Nordgren J., Bergström S., Ernerudh J., Forsberg P., Lindgren P.-E. (2010). Prevalence and diversity of *Borrelia* species in ticks that have bitten humans in Sweden. J. Clin. Microbiol..

[106] Pichon B., Egan D., Rogers M., Gray J. (2003). Detection and identification of pathogens and host DNA in unfed host-seeking *Ixodes ricinus* L. (Acari: Ixodidae). J. Med. Entomol..

[107] Rollend L., Fish D., Childs J.E. (2013). Transovarial transmission of *Borrelia* spirochetes by *Ixodes scapularis*: A summary of the literature and recent observations. Ticks Tick-Borne Dis..

[108] Breuner N.E., Hojgaard A., Replogle A.J., Boegler K.A., Eisen L. (2018). Transmission of the relapsing fever spirochete, *Borrelia miyamotoi*, by single transovarially-infected larval *Ixodes scapularis* ticks. Ticks Tick-Borne Dis..

[109] Krause P.J., Fish D., Narasimhan S., Barbour A.G. (2015). *Borrelia miyamotoi* infection in nature and in humans. Clin. Microbiol. Infect..

[110] Hamer S.A., Hickling G.J., Keith R., Sidge J.L., Walker E.D., Tsao J.I. (2012). Associations of passerine birds, rabbits, and ticks with *Borrelia miyamotoi* and *Borrelia andersonii* in Michigan, U.S.A. Parasites Vectors.

[111] Piesman J., Hicks T.C., Sinsky R.J., Obiri G. (1987). Simultaneous transmission of *Borrelia burgdorferi* and *Babesia microti* by individual nymphal *Ixodes dammini* ticks. J. Clin. Microbiol..

[112] Hofhuis A., van de Kassteele J., Sprong H., van den Wijngaard C.C., Harms M.G., Fonville M., van Leeuwen A.D., Simões M., van Pelt W. (2017). Predicting the risk of Lyme borreliosis after a tick bite, using a structural equation model. PLoS ONE.

[113] Wilhelmsson P., Fryland L., Lindblom P., Sjöwall J., Ahlm C., Berglund J., Haglund M., Henningsson A.J., Nolskog P., Nordberg M. (2016). A prospective study on the incidence of *Borrelia burgdorferi* sensu lato infection after a tick bite in Sweden and on the Åland Islands, Finland (2008–2009). Ticks Tick-Borne Dis..

[114] Wormser G.P., Dattwyler R.J., Shapiro E.D., Halperin J.J., Steere A.C., Klempner M.S., Krause P.J., Bakken J.S., Strle F., Stanek G. (2006). The Clinical assessment, treatment, and prevention of Lyme disease, human granulocytic anaplasmosis, and babesiosis: Clinical practice guidelines by the Infectious Diseases Society of America. Clin. Infect. Dis..

[115] (2020). Babesiosis and Tick-Borne Pathogens Subcommittee Report to the Tick-Borne Disease Working Group. HHS.gov. https://www.hhs.gov/ash/advisory-committees/tickbornedisease/reports/babesiosis-subcomm-2020/index.html.

[116] Dixon D.M., Walker D.H., Walls M., Branda J.A., Clark S., Dumler J.S., Horowitz H.W., Pritt B.S., Sexton D.J., Storch G.A. (2020). Ehrlichiosis and Anaplasmosis Subcommittee Report to the Tick-Borne Disease Working Group. HHS.gov. https://www.hhs.gov/ash/advisory-committees/tickbornedisease/reports/ehrlichiosis-and-anaplasmosis-subcommittee-report-2020/index.html.

[117] Tonnetti L., Dodd R.Y., Foster G., Stramer S.L. (2022). *Babesia* blood testing: The first-year experience. Transfusion.

[118] Bloch E.M., Krause P.J. (2020). Blood screening for *Babesia* in the blood supply. Ann. Blood.

[119] Stanley J., Stramer S.L., Erickson Y., Cruz J., Gorlin J., Janzen M., Rossmann S.N., Straus T., Albrecht P., Pate L.L. (2021). Detection of *Babesia* RNA and DNA in whole blood samples from US blood donations. Transfusion.

[120] Bloch E.M., Krause P.J., Tonnetti L. (2021). Preventing transfusion-transmitted babesiosis. Pathogens.

[121] Krawczyk A.I., Röttjers L., Fonville M., Takumi K., Takken W., Faust K., Sprong H. (2021). Quantitative microbial population study reveals geographical differences in bacterial symbionts of Ixodes ricinus. Microbiome.

[122] Geoghegan J.L., Holmes E.C. (2017). Predicting virus emergence amid evolutionary noise. Open Biol..

[123] Gyllemark P., Wilhelmsson P., Elm C., Hoornstra D., Hovius J.W., Johansson M., Tjernberg I., Lindgren P.-E., Henningsson A.J., Sjöwall J. (2021). Are other tick-borne infections overlooked in patients investigated for Lyme neuroborreliosis? A large retrospective study from South-Eastern Sweden. Ticks Tick-Borne Dis..

[124] Eisen L. (2018). Pathogen transmission in relation to duration of attachment by *Ixodes scapularis* ticks. Ticks Tick-Borne Dis..

[125] Hersh M.H., Ostfeld R.S., McHenry D.J., Tibbetts M., Brunner J.L., Killilea M.E., LoGiudice K., Schmidt K.A., Keesing F. (2014). Co-infection of blacklegged ticks with *Babesia microti* and *Borrelia burgdorferi* is higher than expected and acquired from small mammal hosts. PLoS ONE.

[126] Kurtenbach K., De Michelis S., Sewell H.S., Etti S., Schäfer S.M., Hails R., Collares-Pereira M., Santos-Reis M., Haninçová K., Labuda M. (2001). Distinct combinations of *Borrelia burgdorferi* sensu lato genospecies found in individual questing ticks from Europe. Appl. Environ. Microbiol..

[127] Ginsberg H.S. (2008). Potential effects of mixed infections in ticks on transmission dynamics of pathogens: Comparative analysis of published records. Exp. Appl. Acarol..

[128] Herrmann C., Gern L., Voordouw M.J. (2013). Species co-occurrence patterns among Lyme borreliosis pathogens in the tick vector *Ixodes ricinus*. Appl. Environ. Microbiol..

[129] Kugeler K.J., Schwartz A.M., Delorey M.J., Mead P.S., Hinckley A.F. (2021). Estimating the frequency of Lyme disease diagnoses, United States, 2010–2018. Emerg. Infect. Dis..

[130] Kobayashi T., Higgins Y., Melia M.T., Auwaerter P.G. (2021). Mistaken identity: Many diagnoses are frequently misattributed to Lyme disease. Am. J. Med..

[131] Gray E.B., Herwaldt B.L. (2019). Babesiosis Surveillance—United States, 2011–2015. MMWR Surveill. Summ..

[132] Dammin G.J., Spielman A., Benach J.L., Piesman J. (1981). The rising incidence of clinical *Babesia microti* infection. Hum. Pathol..

[133] Bregnard C., Rais O., Voordouw M.J. (2020). Climate and tree seed production predict the abundance of the European Lyme disease vector over a 15-year period. Parasites Vectors.

[134] Scharlemann J., Johnson P., Smith A., Macdonald D., Randolph S. (2008). Trends in Ixodid tick abundance and distribution in Great Britain. Med. Vet. Entomol..

[135] Vandekerckhove O., De Buck E., Van Wijngaerden E. (2021). Lyme disease in Western Europe: An emerging problem? A systematic review. Acta Clin. Belg..

[136] Public Health Agency of Sweden Årssammanfattning Smittsamma Sjukdomar 2021 [Annual Summary—Infectious Diseases 2021]. https://www.folkhalsomyndigheten.se/folkhalsorapportering-statistik/tolkad-rapportering/arsrapporter-anmalningspliktiga-sjukdomar/arsrapporter-2021/arssammanfattning-2021/.

[137] Public Health Agency of Sweden Tick Borne Encephalitis (TBE)—Sjukdomsstatistik. [Tick-borne Encephalitis (TBE)—Disease Statistics]. https://www.folkhalsomyndigheten.se/folkhalsorapportering-statistik/statistik-a-o/sjukdomsstatistik/tick-borne-encephalitis-tbe/.

[138] Finnish Institute for Health and Welfare Registret för Smittsamma Sjukdomar, Statistisk Databas—THL Användargränssnitt för Data Kuber Och Sammanfattningar. [The Registry for Infectious Diseases, Statistical Database—THL User Interface for Data Cubes and Summaries]. https://sampo.thl.fi/pivot/prod/sv/ttr/shp/fact_shp?row=area-12260&column=time-12059&filter=reportgroup-12465.

[139] Lantos P.M., Wormser G.P. (2014). Chronic coinfections in patients diagnosed with chronic Lyme disease: A systematic review. Am. J. Med..

[140] Hunfeld K.P., Lambert A., Kampen H., Albert S., Epe C., Brade V., Tenter A.M. (2002). Seroprevalence of Babesia infections in humans exposed to ticks in midwestern Germany. J. Clin. Microbiol..

[141] Rigaud E., Jaulhac B., Garcia-Bonnet N., Hunfeld K.P., Féménia F., Huet D., Goulvestre C., Vaillant V., Deffontaines G., Abadia-Benoist G. (2016). Seroprevalence of seven pathogens transmitted by the Ixodes ricinus tick in forestry workers in France. Clin. Microbiol. Infect..

[142] Montero E., Folgueras M., Rodriguez-Pérez M., Pérez-Ls L., Díaz-Arias J., Meana M., Revuelta B., Haapasalo K., Collazos J., Asensi V. (2023). Retrospective study of the epidemiological risk and serological diagnosis of human babesiosis in Asturias, Northwestern Spain. Parasites Vectors.

[143] Nilsson K., Skoog E., Jones V., Labbé Sandelin L., Björling C., Fridenström E., Edvinsson M., Mårtensson A., Olsen B. (2021). A comprehensive clinical and laboratory evaluation of 224 patients with persistent symptoms attributed to presumed tick-bite exposure. PLoS ONE.

[144] Uhnoo I., Cars O., Christensson D., Nyström-Rosander C. (1992). First documented case of human babesiosis in Sweden. Scand. J. Infect. Dis..

[145] Bläckberg J., Lazarevic V.L., Hunfeld K.P., Persson K.E.M. (2018). Low-virulent *Babesia venatorum* infection masquerading as hemophagocytic syndrome. Ann. Hematol..

[146] Mørch K., Holmaas G., Frolander P.S., Kristoffersen E.K. (2015). Severe human *Babesia divergens* infection in Norway. Int. J. Infect. Dis..

[147] Thortveit E.T., Aase A., Petersen L.B., Lorentzen Å.R., Mygland Å., Ljøstad U. (2020). Human seroprevalence of antibodies to tick-borne microbes in southern Norway. Ticks Tick-Borne Dis..

[148] Kallio E.R., Begon M., Birtles R.J., Bown K.J., Koskela E., Mappes T., Watts P.C. (2014). First report of *Anaplasma phagocytophilum* and *Babesia microti* in rodents in Finland. Vector Borne Zoonotic Dis..

[149] Hasle G., Leinaas H.P., Roed K.H., Oines O. (2011). Transport of *Babesia venatorum*-infected *Ixodes ricinus* to Norway by northward migrating passerine birds. Acta Vet. Scand..

[150] Young K.M., Corrin T., Wilhelm B., Uhland C., Greig J., Mascarenhas M., Waddell L.A. (2019). Zoonotic *Babesia*: A scoping review of the global evidence. PLoS ONE.

[151] Ursinus J., Vrijmoeth H.D., Harms M.G., Tulen A.D., Knoop H., Gauw S.A., Zomer T.P., Wong A., Friesema I.H., Vermeeren Y.M. (2021). Prevalence of persistent symptoms after treatment for Lyme borreliosis: A prospective observational cohort study. Lancet Reg. Health-Eur..

[152] Sloupenska K., Dolezilkova J., Koubkova B., Hutyrova B., Racansky M., Horak P., Golovchenko M., Raska M., Rudenko N., Krupka M. (2021). Seroprevalence of antibodies against tick-borne pathogens in Czech patients with suspected post-treatment Lyme disease syndrome. Microorganisms.

[153] Krause P.J., Spielman A., Telford S.R., Sikand V.K., McKay K., Christianson D., Pollack R.J., Brassard P., Magera J., Ryan R. (1998). Persistent parasitemia after acute babesiosis. N. Engl. J. Med..

[154] Bloch E.M., Kumar S., Krause P.J. (2019). Persistence of *Babesia microti* infection in humans. Pathogens.

[155] Lantos P.M., Rumbaugh J., Bockenstedt L.K., Falck-Ytter Y.T., Aguero-Rosenfeld M.E., Auwaerter P.G., Baldwin K., Bannuru R.R., Belani K.K., Bowie W.R. (2021). Clinical Practice Guidelines by the Infectious Diseases Society of America (IDSA), American Academy of Neurology (AAN), and American College of Rheumatology (ACR): 2020 Guidelines for the Prevention, Diagnosis, and Treatment of Lyme Disease. Arthritis Care Res..

[156] Martínez-Balzano C., Hess M., Malhotra A., Lenox R. (2015). Severe babesiosis and *Borrelia burgdorferi* co-infection. QJM Int. J. Med..

[157] Bobe J.R., Jutras B.L., Horn E.J., Embers M.E., Bailey A., Moritz R.L., Zhang Y., Soloski M.J., Ostfeld R.S., Marconi R.T. (2021). Recent Progress in Lyme disease and remaining challenges. Front. Med..

[158] Mowla S.J., Drexler N.A., Cherry C.C., Annambholta P.D., Kracalik I.T., Basavaraju S.V. (2021). Ehrlichiosis and Anaplasmosis among transfusion and transplant recipients in the United States. Emerg. Infect. Dis..

[159] Centers for Disease Control and Prevention (CDC) (2008). Anaplasma phagocytophilum transmitted through blood transfusion--Minnesota, 2007. MMWR Morb. Mortal. Wkly. Rep..

[160] Herwaldt B.L., Linden J.V., Bosserman E., Young C., Olkowska D., Wilson M. (2011). Transfusion-associated babesiosis in the United States: A description of cases. Ann. Intern. Med..

[161] Krause P.J., Hendrickson J.E., Steeves T.K., Fish D. (2015). Blood transfusion transmission of the tick-borne relapsing fever spirochete *Borrelia miyamotoi* in mice. Transfusion.

[162] Hildebrandt A., Gray J.S., Hunfeld K.P. (2013). Human babesiosis in Europe: What clinicians need to know. Infection.

[163] Krause P.J., Gewurz B.E., Hill D., Marty F.M., Vannier E., Foppa I.M., Furman R.R., Neuhaus E., Skowron G., Gupta S. (2008). Persistent and relapsing babesiosis in immunocompromised patients. Clin. Infect. Dis. Off. Publ. Infect. Dis. Soc. Am..

[164] Goel R., Westblade L.F., Kessler D.A., Sfeir M., Slavinski S., Backenson B., Gebhardt L., Kane K., Laurence J., Scherr D. (2018). Death from transfusion-transmitted anaplasmosis, New York, USA, 2017. Emerg. Infect. Dis..

[165] Levin A.E., Krause P.J. (2016). Transfusion-transmitted babesiosis: Is it time to screen the blood supply?. Curr. Opin. Hematol..

[166] Fong I.W. (2020). Blood transfusion-associated infections in the twenty-first century: New challenges. Current Trends and Concerns in Infectious Diseases.

[167] Fiecek B., Matławska M., Gołąb E., Chmielewski T. (2020). Risk of transmission of tick-borne diseases by blood transfusion. Postępy Mikrobiol-Adv. Microbiol..

[168] Goodell A.J., Bloch E.M., Krause P.J., Custer B. (2014). Costs, consequences, and cost-effectiveness of strategies for Babesia microti donor screening of the US blood supply. Transfusion.

